# Leaf Blight in *Ilex verticillata* Caused by *Alternaria alternata*: Mechanisms of Antioxidant Defense, Phytohormone Crosstalk, and Oxidative Stress Responses

**DOI:** 10.3390/plants14193057

**Published:** 2025-10-03

**Authors:** Huijie Lu, Caixia Zhou, Peiwen Cheng, Liangye Huang, Qinyuan Shen, Ye Zheng, Yihui Li, Wenjun Dai, Jianhong Zhang, Dengfeng Shen, Anket Sharma, Muhammad Junaid Rao, Bingsong Zheng, Huwei Yuan

**Affiliations:** 1 National Key Laboratory for Development and Utilization of Forest Food Resources, Zhejiang A&F University, Hangzhou 311300, China; 19857999948@163.com (H.L.); 2022602122157@stu.zafu.edu.cn (C.Z.); chengpeiwen0826@163.com (P.C.); 2023602121036@stu.zafu.edu.cn (L.H.); 2024202011016@stu.zafu.edu.cn (Q.S.); 2024102012044@stu.zafu.edu.cn (Y.Z.); 2024602122054@stu.zafu.edu.cn (Y.L.); 2023602122015@stu.zafu.edu.cn (W.D.); anketsharma@gmail.com (A.S.); 2 Provincial Key Laboratory for Non-Wood Forest and Quality Control and Utilization of Its Products, Zhejiang A&F University, Hangzhou 311300, China; 3 Ningbo Key Laboratory of Characteristic Horticultural Crops in Quality Adjustment and Resistance Breeding, Ningbo Academy of Agricultural Sciences, Ningbo 315041, China; nbjianhong@163.com (J.Z.); dengfengdz@163.com (D.S.)

**Keywords:** *Alternaria alternata*, *Ilex verticillata*, leaf blight, host–pathogen interaction, transcriptomics, antioxidant defense, phytohormones, oxidative stress

## Abstract

*Ilex verticillata* (winterberry) is a valuable ornamental shrub increasingly threatened by leaf blight, a disease that compromises its aesthetic and economic value. While fungal pathogens like *Alternaria alternata* are known to cause leaf blight in horticultural crops, their role in *I. verticillata* and the host’s defense mechanisms have not been fully characterized. Our study investigated the pathogen-host interaction by identifying the causal agent and examining the physiological and molecular defense mechanisms of *I. verticillata*. Through morphological and multi-locus molecular analyses (ITS, TEF1-α, G3PDH, RPB2), *A. alternata* was confirmed as the primary pathogen, fulfilling Koch’s postulates. Pathogenicity assays revealed distinct disease progression stages, from necrotic lesions to tissue degradation. Transcriptomic profiling uncovered dynamic host responses, with early upregulation of pattern recognition receptors (PRRs) and transcripts encoding antioxidant enzymes (SOD, CAT), followed by downregulation of metabolic pathway genes. Phytohormone analysis highlighted intricate crosstalk, with salicylic acid (SA) peaking during mid-infection and jasmonic acid (JA) rebounding later, reflecting a coordinated defense strategy. Additionally, the oxidative stress marker malondialdehyde (MDA), an indicator of membrane lipid peroxidation, surged early, indicating membrane damage, while sustained induction of antioxidant enzymes suggested adaptive responses. The key finding was distinct phytohormone crosstalk, characterized by a mid-infection SA peak followed by a late JA rebound, alongside an early oxidative burst marked by MDA accumulation and sustained antioxidant enzyme activity. These findings provide a framework for understanding *I. verticillata’s* defense mechanisms and offer insights for developing targeted disease management strategies, such as resistant cultivar breeding or hormone-mediated interventions.

## 1. Introduction

*Ilex verticillata* (winterberry) is a deciduous shrub native to the northeastern United States [1], valued in horticulture for its persistent, vibrant red berries [2]. Introduced to China in 2006, it is now cultivated for landscaping and slope stabilization [1,2]. However, successful establishment outside its native range is challenged by adaptation issues, including significant susceptibility to pests and diseases such as spider mites, grey mold, and notably, leaf blight, which compromises its aesthetic and economic value [3,4]. However, field observations indicate that leaf blight causes severe foliar damage and fruit loss, potentially leading to plant death and constraining the cultivation of this valuable species [5].

Leaf blight, often favored by warm, humid conditions [6], is frequently caused by fungal pathogens in the genus *Alternaria* (species *A. alternata*, *A. tenuissima*, etc.) [7,8,9]. Fungal pathogens in the genus *Alternaria* are notorious for their broad host range, which includes numerous ornamental species (lilies and poplar), highlighting a significant threat to horticultural crops [10,11,12,13]. These fungi produce toxins that disrupt cellular functions, leading to lesion formation and tissue decay. Similarly, *Colletotrichum* species, such as *C. siamense*, have been identified as pathogens causing leaf blight in *I. verticillata* [14]. *Alternaria alternata*, a ubiquitous species, is particularly destructive, inducing leaf spots, blight, and rot through the production of phytotoxins and enzymatic degradation of host tissues [10,11]. While *A. alternata* has been implicated in leaf blight of other *Ilex* species and related plants, its role in *I. verticillata* leaf blight has not been definitively established. Accurate pathogen identification, coupled with an understanding of its interaction with the host, is critical for developing targeted control measures.

Plants defend against necrotrophic pathogens like *Alternaria* spp. through an integrated response involving structural reinforcement, antioxidant systems, and phytohormone signaling [15,16,17,18]. Early infection often triggers oxidative burst, leading to membrane damage marked by lipid peroxidation and malondialdehyde (MDA) accumulation [19], as observed in *Osmanthus fragrans* and *Phoebe zhennan* following *Alternaria* challenge [20,21]. To mitigate oxidative stress, plants activate their antioxidant enzymes such as superoxide dismutase (SOD), peroxidase (POD), and catalase (CAT) [13,22,23,24], though prolonged infection may suppress their activity [25]. Simultaneously, pathogen recognition via pattern recognition receptors (PRRs) can initiate a hypersensitive response (HR) and reinforce structural defenses through secondary metabolites like phenylpropanoids and pathogenesis-related (PR) proteins, including phenylalanine ammonia-lyase (PAL) and chitinase (CHI) [18,26,27]. At the molecular level, Transcriptomic studies in chrysanthemum and tree peony (*Paeonia suffruticosa* Andrews) have further highlighted the importance of jasmonic acid (JA) and salicylic acid (SA) pathways in regulating defense against *A. alternata* [28,29], with key regulators involved in JA signaling such as JAZ and MYC2 [28,29,30,31]. Similarly, WRKY and NAC transcription factors are differentially expressed in populus and tobacco (*Nicotiana attenuata*), in response to *A. alternata* infection [32,33]. However, despite these advances in related species, the defense response of *I. verticillata* to *A. alternata* remains uncharacterized. Examining these mechanisms in winterberry may identify key resistance traits applicable to breeding programs.

However, in contrast to well-studied pathosystems like *Alternaria*-tomato or *Alternaria*-apple tissues [10,11], the causal agent of leaf blight in *I. verticillata* and the host’s defense responses are not well-defined [1,2]. This knowledge gap hinders the development of targeted and sustainable management strategies. Therefore, this study had three objectives: (1) to isolate and identify the causal agent of leaf blight in *I. verticillata* ‘Meister’ through morphological, molecular, and pathogenicity analyses; (2) to characterize the host’s physiological response by measuring oxidative stress markers and antioxidant enzyme activities; and (3) to profile the transcriptional changes during infection to identify key defense-related pathways.

## 2. Results

### 2.1. Isolation and Purification of the Pathogen Causing Holly Leaf Blight

**Figure 1:**
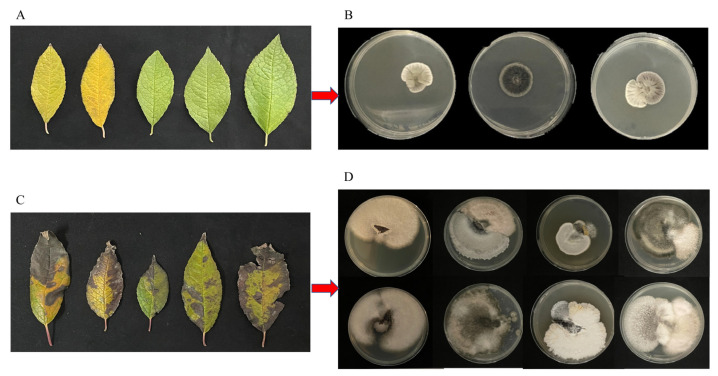
Isolation of the pathogen of leaf blight disease in *I. verticillate*. (**A**): Healthy leaves, (**B**): Tissue isolation results of healthy leaves, (**C**): Diseased leaves, (**D**): Tissue isolation results of diseased leaves.

Healthy *I. verticillata* leaves showed no symptoms (Figure 1A) and yielded no pathogens (Figure 1B). In contrast, diseased leaves exhibited black spots, curling, and desiccation (Figure 1C). From these symptomatic tissues, we isolated three distinct fungal strains (Y9-2-1, Y10, Y16; Figure 1D). The clear contrast between healthy and diseased samples identified these isolates as putative causative agents. Subsequent purification enabled morphological and molecular characterization to identify the leaf blight pathogens.

### 2.2. Morphological Characteristics and Phylogenetic Analysis of Whorled Holly Leaf Blight Pathogen

**Figure 2:**
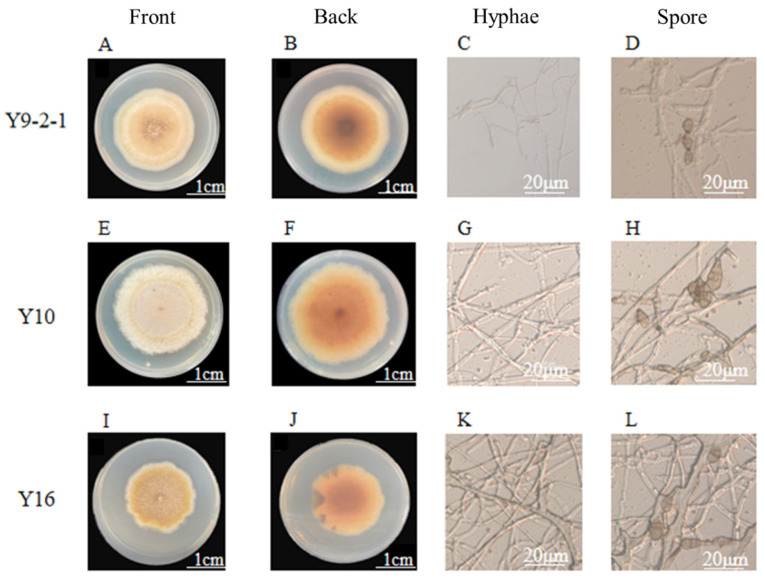
Cultural and morphological characteristics of *Alternaria* isolates causing leaf blight disease of *I. verticillata*. (**A**,**E**,**I**) Front view of colonies for strains Y9-2-1, Y10, and Y16, respectively, showing dense aerial hyphae with neat margins. (**B**,**F**,**J**) Reverse side of colonies displaying yellow-brown pigmentation in early growth, darkening to brown or black with maturity. (**C**,**G**,**K**) Microscopic features of hyphae. (**D**,**H**,**L**) Conidia observed singly or in chains, exhibiting smooth surfaces, distinct transverse and longitudinal septa, and an inverted club-shaped to ovoid morphology. The high degree of morphological similarity among the three isolates supports their preliminary identification as *Alternaria* sp. and indicates the same strain. Scale bars: 1 cm and 20 µm.

Morphological analysis revealed that strains Y9-2-1, Y10, and Y16 shared similar cultural and microscopic features (Figure 2). Colonies initially displayed yellow-brown pigmentation on the reverse side (Figure 2B,F,J), darkening to brown or black with maturity. They exhibited dense aerial hyphae (Figure 2A,E,I) and orderly marginal growth. Conidiophores were solitary or clustered, and light to dark brown in color (Figure 2D,H,L). Conidia were observed singly or in chains, with smooth surfaces, distinct transverse and longitudinal septa, and an inverted club-shaped to ovoid morphology (Figure 2C,G,K). The high degree of morphological similarity among isolates Y9-2-1, Y10, and Y16 supported their preliminary identification as *Alternaria* sp. and indicated they likely represent the same strain.

**Figure 3:**
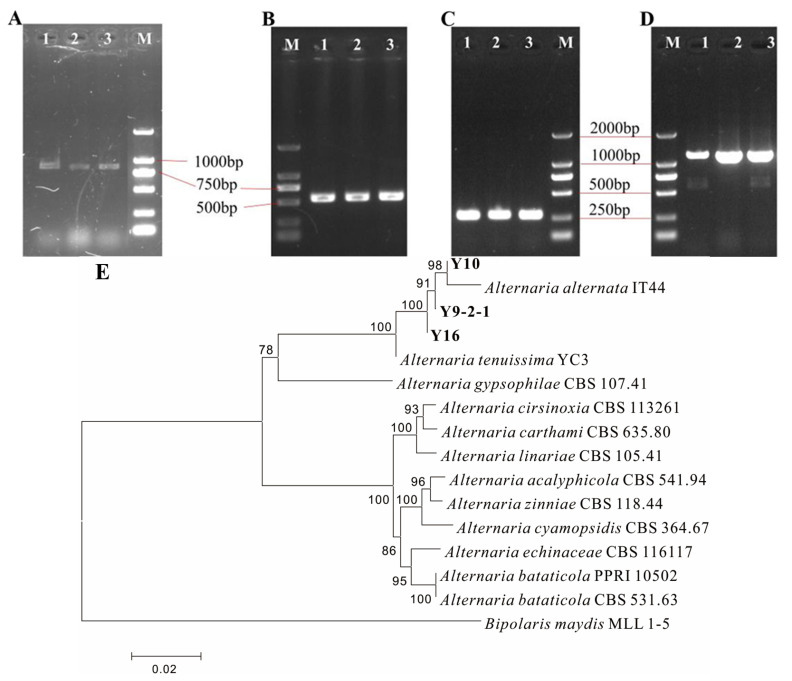
Molecular identification and phylogenetic analysis of *Alternaria* isolates causing leaf blight disease of *I. verticillate*. (**A**–**D**) Agarose gel electrophoresis of PCR amplicons for the (**A**) G3PDH, (**B**) ITS, (**C**) TEF1-α, and (**D**) RPB2 gene regions. Lanes: 1, isolate Y9-2-1; 2, isolate Y10; 3, isolate Y16; M, DL2000 DNA Marker. (**E**): Phylogenetic tree constructed from the concatenated sequences of the ITS, TEF1-α, RPB2, and G3PDH loci using the Neighbor-Joining method. The numbers at the nodes represent bootstrap percentages based on 1000 replicates; only values ≥ 50% are shown. The scale bar represents 0.02 nucleotide substitutions per site. Bold characters represent the strain isolated in *I. verticillate*.

Phylogenetic analysis based on four genetic markers (G3PDH ~800 bp, ITS ~500 bp, TEF1-α ~250 bp, RPB2 ~1100 bp) were used to identify the fungal isolates and all isolates produced PCR amplicons of the expected sizes (800 bp, 500 bp, 250 bp, 1100 bp, respectively) for *Alternaria* species (Figure 3A–D). The concatenated sequence analysis placed isolates Y9-2-1, Y10, and Y16 within a strongly supported clade (100% bootstrap) alongside reference strains of *A. alternata* (Figure 3E). The high sequence similarity across all four loci and their consistent phylogenetic placement provide conclusive evidence for identifying the pathogen as *A. alternata*. Furthermore, the identical sequences for each locus among the three isolates indicate they represent a single strain responsible for the leaf blight symptoms on *I. verticillata*.

### 2.3. Pathogenicity Assessment of Alternaria alternata on Ilex verticillata

**Figure 4:**
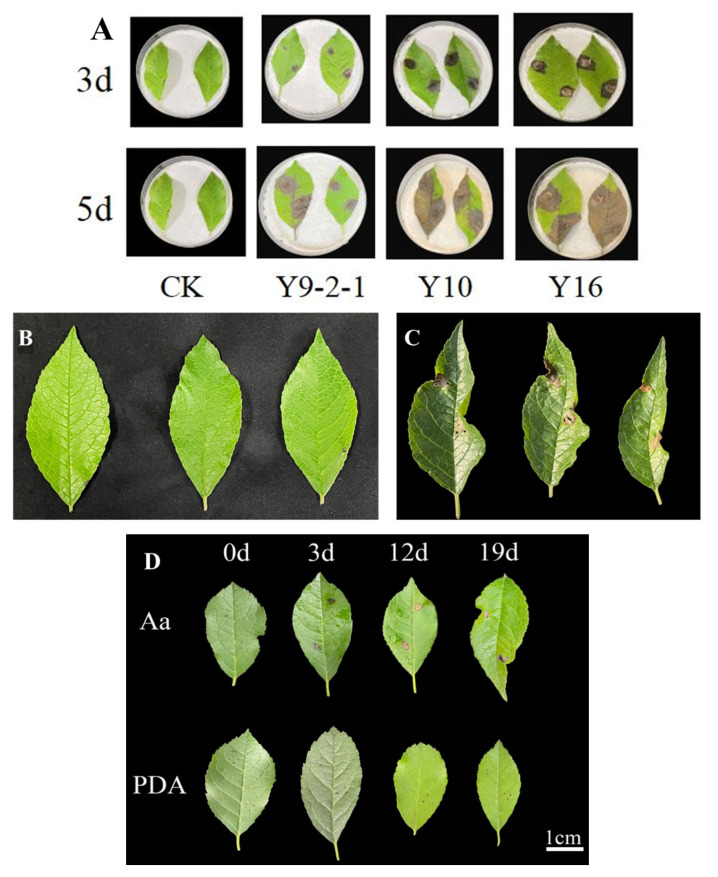
Disease progression, pathogenicity, and temporal progression on *I. verticillata* leaves following inoculation with *Alternaria alternata*. (**A**): *A. alternata* isolates Y9-2-1, Y10, and Y16 inoculation compared to non-inoculated control (CK). Photographs were taken at 3 days (3 d) and 5 days (5 d) post-inoculation, showing characteristic leaf blight symptoms including necrotic lesions and chlorosis. (**B**): Healthy, non-inoculated control leaves showing normal morphology. (**C**): Diseased leaves at 5 days post-inoculation with *A. alternata* isolates, exhibiting severe blight symptoms including necrotic lesions, chlorotic halos, and tissue collapse. (**D**): Temporal progression of *Alternaria alternata* infection on *I. verticillata* leaves. Leaves were inoculated with either sterile potato dextrose agar medium (PDA, control) or *A. alternata* conidial suspension (Aa). Initial symptoms (3 dpi) appeared as small necrotic spots that expanded into coalescing lesions with chlorotic halos by 12 dpi, culminating in extensive tissue necrosis by 19 dpi. PDA controls showed no pathological changes throughout the observation period.

Our detached-leaf assays showed *A. alternata* caused distinct necrotic lesions within 3 days (Figure 4A). By day 5, the disease had progressed to 100% incidence, with lesions expanding to the entire leaf surface. The identity of the pathogen was confirmed by re-isolating fungi from the diseased tissue, which was morphologically identical to the original inoculant and confirmed as *A. alternata* by molecular analysis (Supplementary Figure S1A,B), thereby fulfilling Koch’s postulates. Figure 4B shows healthy control leaves. Potted plant trials mirrored natural infections. Initial 2–5 mm black lesions expanded with characteristic leaf curling (Figure 4C), progressing to tissue collapse and perforation, identical to field symptoms. The consistent disease development across all replicates in both detached-leaf and potted plant assays demonstrates the pathogenicity of *A. alternata* in *I. verticillata*. The congruence of results from these complementary experimental systems provides strong evidence supporting *A. alternata* as the causative agent of leaf blight.

In inoculated ‘Meister’ seedlings, *A. alternata* infection progressed through distinct stages (Figure 4D). Small black necrotic spots (1–3 mm) appeared by 3 dpi, expanding into rusty brown lesions with leaf curling by 12 dpi. By 19 dpi, severe tissue degradation caused dry, perforated lesions. This clear progression from localized spots to widespread damage confirms the pathogen’s aggressive colonization pattern in whorled holly.

### 2.4. Transcriptomic Profiling of Host Responses During Alternaria alternata Infection

#### 2.4.1. Global Differential Gene Expression Patterns

**Figure 5:**
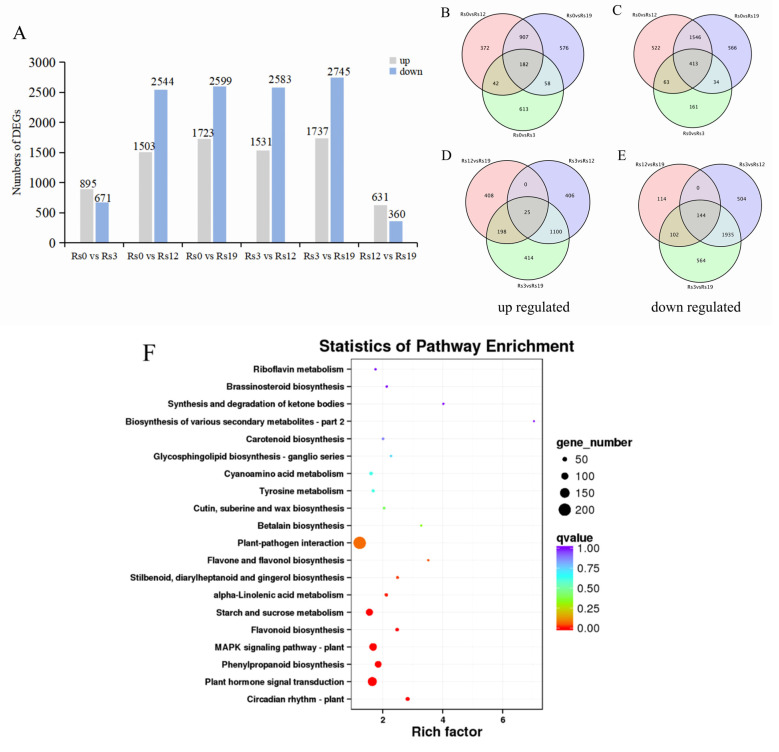
Temporal dynamics of DEGs (FDR < 0.05 and |log~2~Fold Change| ≥ 1) in *Ilex verticillata* leaves during *Alternaria alternata* infection. (**A**): Histogram showing the total number of DEGs identified at 3-, 12-, and 19 days post-inoculation (dpi) compared to uninoculated controls (0 dpi). (**B**,**C**) Venn diagrams illustrating (**B**) upregulated and (**C**) downregulated DEGs at each time point relative to 0 dpi controls, revealing stage-specific transcriptional responses. (**D**,**E**) Comparative Venn analyses of (**D**) upregulated and (**E**) downregulated DEGs across all infection timepoints (3 vs. 12 vs. 19 dpi), highlighting conserved and unique gene expression patterns during disease progression. (**F**): Top 20 significantly enriched KEGG pathways (FDR < 0.05) during *A. alternata* infection. The Rich Factor (x-axis) is the ratio of the number of DEGs to the total number of genes in a given pathway. The size of the bubble corresponds to the number of DEGs assigned to the pathway, and the color represents the -log10 transformed *p*-value from the enrichment analysis.

RNA-seq revealed dramatic gene expression changes in *I. verticillata* leaves during *A. alternata* infection (Figure 5). Differentially expressed genes (DEGs) were identified using DESeq2 with a threshold of false discovery rate (FDR) < 0.05 and |log~2~Fold Change| ≥ 1. Using these criteria, we observed 1566 DEGs at 3 dpi (895 up- and 671 down-regulated), increasing to 4047 DEGs by 12 dpi (1503 up- and 2544 down-regulated) and 4322 DEGs at 19 dpi (1723 up- and 2599 down-regulated) (Figure 5A). This shifts from early upregulation (stress response) to late-stage downregulation (plant hormone signal transduction; Supplementary Figure S2). Comparing timepoints revealed major transcriptional shifts: 12 dpi introduced 1531 new upregulated and 2583 downregulated genes versus 3 dpi, while 19 dpi added 1737 up- /2745 down-regulated DEGs. Late-stage changes (12 vs. 19 dpi) were more moderate (631↑/360↓).

#### 2.4.2. Stage-Specific Transcriptional Signatures

Venn analyses identified stage-specific responses (Figure 5B–E). Early infection featured 42 consistently upregulated and 63 downregulated genes (3–12 dpi), while late infection shared 907 up- /1546 down-regulated DEGs (12–19 dpi). Notably, 198 upregulated and 102 downregulated genes maintained their expression from early through mid-infection, suggesting their potential involvement in defense responses. The complete lack of overlapping DEGs between 3 and 19 dpi (using 12 dpi as reference) indicates a wholesale transcriptional reprogramming as infection progresses.

#### 2.4.3. KEGG Enrichment Analysis of Differential Gene Expression

KEGG analysis revealed *I. verticillata’s* coordinated defense against *A. alternata* through three key strategies (Figure 5F): First, pathogen recognition via Plant-pathogen interaction pathways (28 DEGs). Second, intracellular signaling through MAPK (19 DEGs) and hormone transduction (34 DEGs). Third, the production of antimicrobial compounds was activated, as indicated by the enrichment of the Phenylpropanoid and Flavonoid biosynthesis pathways, which are involved in synthesizing defensive secondary metabolites. Concurrently, the plant reshaped its primary metabolism, with changes in Starch and sucrose metabolism potentially indicating a resource reallocation to defense. The enrichment of the α-Linolenic acid pathway, a precursor for jasmonic acid, confirmed the activation of JA-mediated signaling. Furthermore, the induction of Cutin, suberine, and wax biosynthesis suggested a reinforcement of physical barriers at the leaf surface. Lastly, the modulation of Brassinosteroid pathways pointed to a complex hormonal coordination of the overall defense strategy. This multi-tiered response mirrors known plant defense mechanisms: rapid pathogen detection triggers signaling cascades that mobilize both chemical defenses (antimicrobials) and physical barriers (waxes). The conserved MAPK and jasmonate pathways highlight evolutionary convergence in fungal defense strategies. Together, these pathways reveal how whorled holly reconfigures its physiology to detect, signal, and combat fungal invasion at multiple levels.

### 2.5. Transcriptional Dynamics of Plant-Pathogen Interaction Genes During Infection

**Figure 6:**
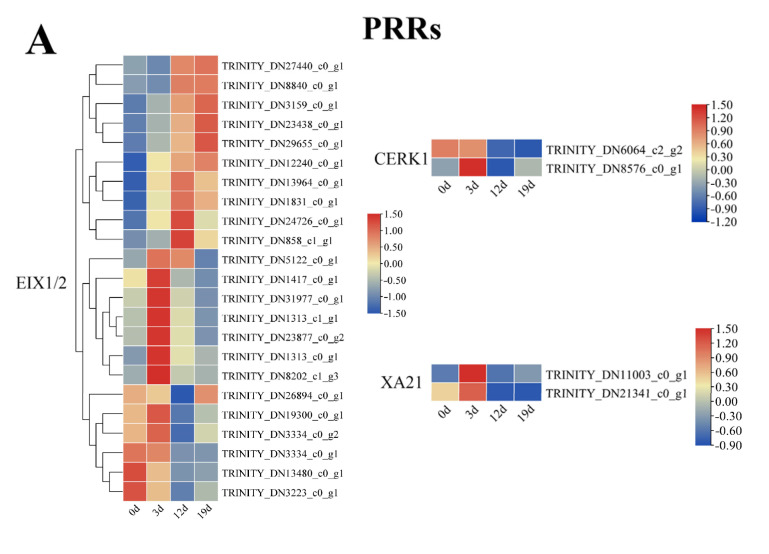

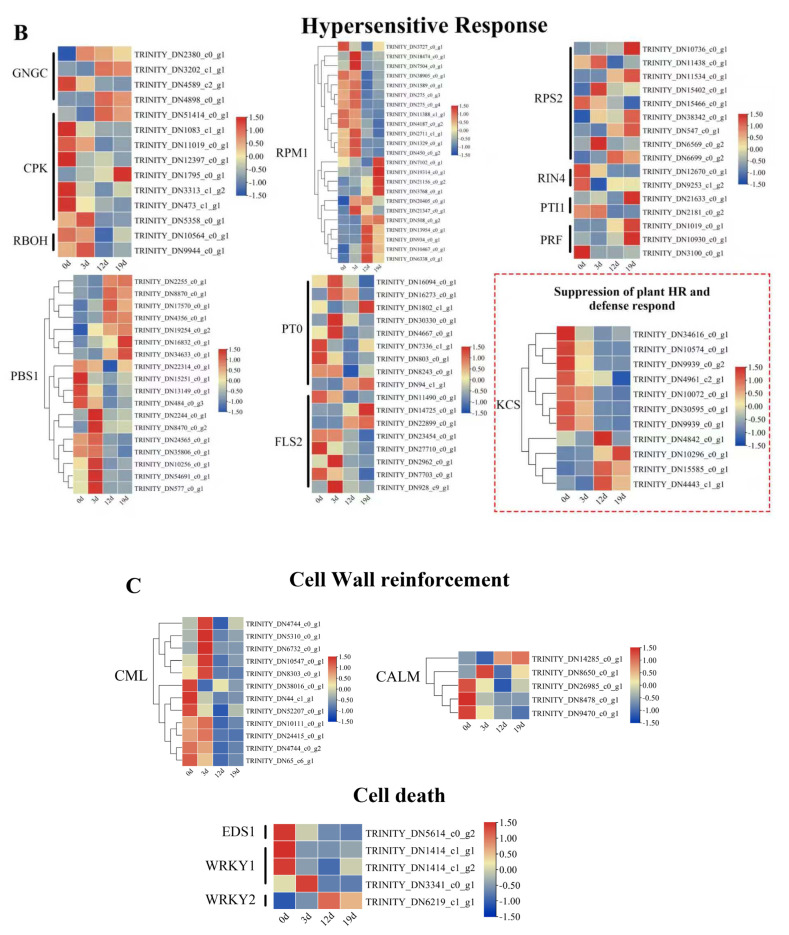

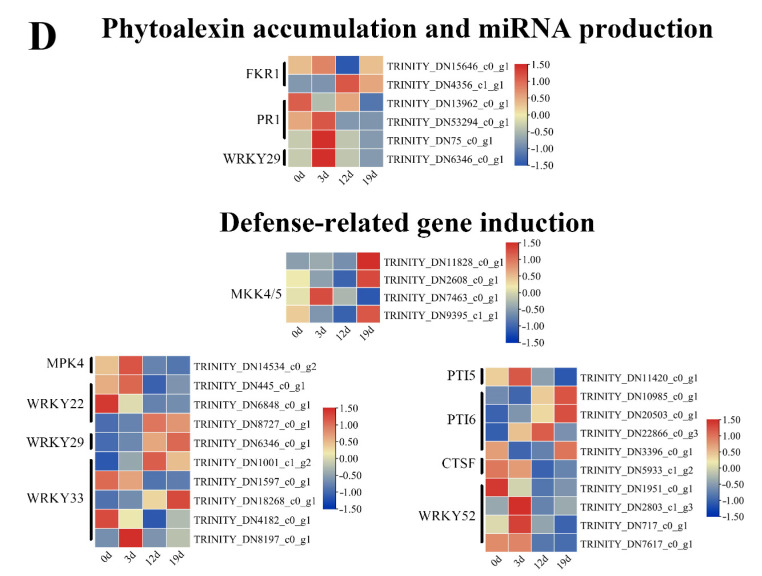
Expression analysis of DEGs (FDR < 0.05, |log2Fold Change| ≥ 1) related to the plant-pathogen interaction pathway. Heatmaps show the Z-score normalized expression of genes that were significantly differentially expressed (False Discovery Rate, FDR < 0.05 and |log2Fold Change| ≥ 1) at one or more time points during *A. alternata* infection (3, 12, 19 dpi) compared to the uninoculated control (0 dpi). (**A**): DEGs encoding pattern recognition receptors (PRRs). (**B**): DEGs associated with the hypersensitive response (HR). (**C**): DEGs involved in cell wall reinforcement and cell death regulation. (**D**): DEGs related to defense-related gene induction, phytoalexin accumulation, and miRNA production.

*I. verticillata* initiate defense against *A. alternata* through precisely timed gene regulation (Figure 6A–C). Key pattern recognition receptors showed phased activation—CERK1 and XA21 peaked early (3 dpi), while specific EIX1/2 genes gradually increased until 12–19 dpi (Figure 6A). This sequential PRR deployment suggests an evolving detection strategy matching pathogen progression. Hypersensitive response genes exhibited complex temporal patterns (Figure 6B). Calcium signaling components (CNGC/CPK) and R-protein regulators (RPM1) showed opposing trends, while the HR inhibitor KCS was consistently suppressed, a pattern that may facilitate the progression of cell death. Early responders like PBS1, PTI0, and FLS2 were rapidly induced and then downregulated, marking the transition from detection to defense execution. Genes associated with structural defenses also showed nuanced regulation, particularly those involved in calcium-mediated signaling (Figure 6C). Calcium signaling components, such as calmodulin-like proteins (CMLs) and calmodulins (CALMs), displayed divergent responses: seven CMLs and one CALM were transiently induced during early infection (0–3 dpi), potentially contributing to the initial activation of defense responses. In contrast, five CMLs and three CALMs were suppressed, while a single CALM maintained elevated expression, suggesting a complex and finely tuned modulation of calcium signals to orchestrate downstream defense processes. Cell death regulators indicated a carefully managed hypersensitive response. WRKY2, a transcription factor often associated with promoting programmed cell death, remained active until 12 dpi before declining. Conversely, EDS1, a central regulator of salicylic acid (SA)-mediated resistance and cell death, along with most WRKY1 homologs, was suppressed, which may serve to limit excessive tissue damage. One WRKY1 variant showed early activation followed by suppression, hinting at isoform-specific roles in the defense response. These patterns reveal a multi-layered defense strategy: (1) Immediate pathogen detection through phased PRR activation, (2) Precise control of hypersensitive response via opposing regulators, and (3) Dynamic modulation of structural defenses and cell death pathways. The coordinated yet flexible gene regulation allows *I. verticillata* to balance effective defense with resource conservation during prolonged infection.

Early infection (0–3 dpi) triggered transient phytoalexin genes (FKR1, PR1, WRKY29), while defense signaling components (MKK4/5, MPK4, PT6) remain active longer (Figure 6D). This dual strategy balances immediate chemical defenses with sustained signaling, helping *I. verticillata* combat initial infection while maintaining resistance as the pathogen advances.

### 2.6. Dynamic Changes in Endogenous Hormone Levels During Pathogen Infection

#### 2.6.1. Variation in Stress-Related Phytohormones During Pathogenesis

**Figure 7:**
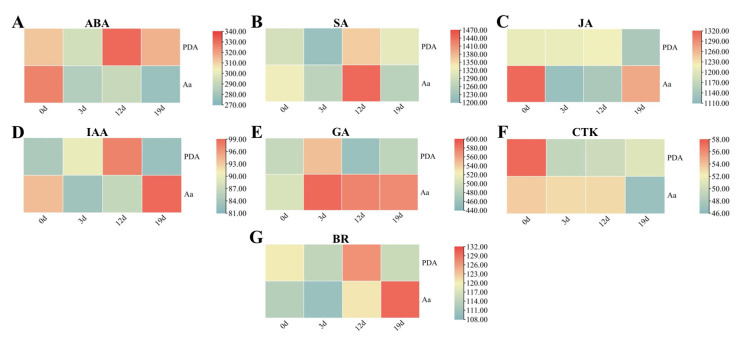
Changes in endogenous hormone levels in *Ilex verticillata* leaf during infection with the leaf blight pathogen. (**A**): ABA content (μg·g^−1^ FW). (**B**): SA content (pmol·g^−1^ FW). (**C**): JA content (pmol·g^−1^ FW). (**D**): IAA content (μg·g^−1^ FW). (**E**): GA content (pg·mL^−1^ FW). (**F**): CTK content (μg·L^−1^ FW). (**G**): BR content (ng·L^−1^ FW). The absolute hormone concentrations and mean values (*n* = 3) for each time point are provided in Supplementary Table S2. The gradient color from blue to red indicates the hormone content from small to large. PDA: *I. verticillata* leaves inoculated with sterile potato dextrose agar medium; Aa: *I. verticillata* leaves inoculated with *A. alternata*; 0 d, 3 d, 12 d, 19 d means 0, 3, 12, 19 days after infection. Note: Hormone levels were quantified using enzyme-linked immunosorbent assay (ELISA). Concentrations are reported in the specific units defined by the standard curves of the respective commercial ELISA kits used for each hormone; therefore, absolute values are not directly comparable across different hormones. The relative changes within each hormone over time are the critical metric.

*I. verticillata* showed dynamic hormone changes during infection (Figure 7A–C). ABA decreased 12.6% by 3 dpi, partially recovering later. SA peaked at 12 dpi (+8.8%; for absolute values see Supplementary Table S2), then dropped sharply, suggesting transient resistance activation. JA showed opposite trends to SA, declining initially but rebounding by 19 dpi. These coordinated, opposing patterns reveal sophisticated hormonal crosstalk regulating defense strategies against *A. alternata*.

#### 2.6.2. Modulation of Growth-Related Hormones During Pathogenesis

*A. alternata* infection disrupted growth-related hormones in *I. verticillata* (Figure 7D–G). Auxin showed a V-shaped pattern, decreasing 7.9% by 12 dpi before rebounding 14.3% above mid-infection levels. GA peaked early (16.7% increase at 3 dpi) and remained elevated (+13.6% at 19 dpi). CTK levels remained stable initially but dropped sharply (11.6%) during late infection (12–19 dpi), potentially regulating senescence. BR displayed unique dynamics—increasing 7.4% in infected plants while decreasing in controls, suggesting pathogen manipulation of host defenses. These coordinated changes reveal: (1) Early GA-mediated growth responses; (2) Late-stage CTK-linked senescence; (3) Pathogen-induced BR manipulation; and (4) Recovery of auxin signaling. The distinct temporal patterns demonstrate how *A. alternata* alters host growth physiology while *I. verticillata* attempts to rebalance its hormonal defenses throughout infection progression.

### 2.7. Oxidative Stress Responses During Pathogen Infection

**Figure 8:**
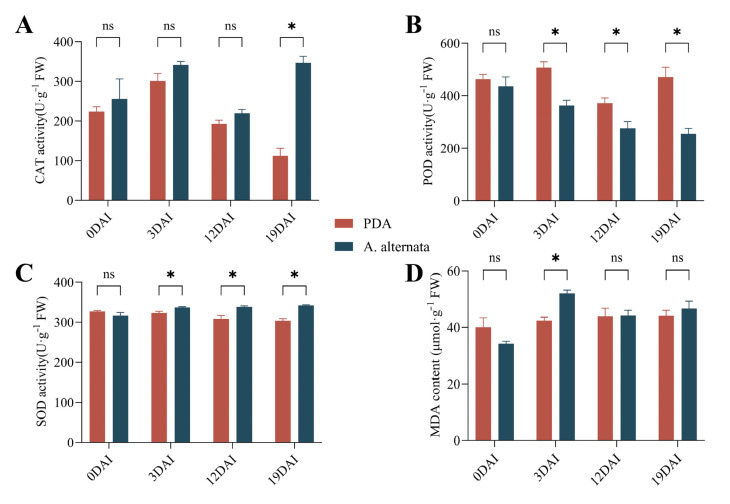
Changes in antioxidant defense enzyme activity and MDA content in leaves of *I. verticillata* at different stages after infection. (**A**): CAT activity. (**B**): POD activity. (**C**): SOD activity. (**D**): MDA content. DAI: Days after infection. PDA: *I. verticillata* leaves inoculated with sterile potato dextrose agar medium; *A. alternata*: *I. verticillata* leaves inoculated with *A. alternata*; 0DAI, 3DAI, 12DAI, 19DAI means 0, 3, 12, 19 days after infection; * represents significant difference at *p* < 0.05 level; ns means no significant difference.

*A. alternata* infection triggered distinct antioxidant responses in *I. verticillata* (Figure 8A–C). CAT activity was generally elevated in infected leaves, and a significant, >3-fold increase was observed at 19 dpi compared to the control (*p* < 0.05, Duncan’s test, n = 3). Conversely, POD activity was consistently suppressed, with maximum reduction at 3 dpi. SOD showed rapid early induction (0–3 dpi) and sustained high activity. This coordinated response suggests SOD acts as the primary ROS scavenger during initial infection, while CAT provides long-term protection. The inverse relationship between POD suppression and SOD/CAT activation indicates a strategic reallocation of antioxidant defenses. MDA levels spiked early (0–3 dpi), confirming significant oxidative membrane damage during initial colonization (Figure 8D). The subsequent MDA decline coincided with sustained high CAT/SOD activities, suggesting either: (1) Successful ROS neutralization by these enzymes, or (2) Activation of membrane repair systems. These findings reveal *I. verticillata*’s dynamic oxidative stress management—rapid SOD induction for immediate ROS quenching, supported by persistent CAT activity for prolonged protection, while POD appears less critical in this pathosystem.

## 3. Discussion

The findings of this study provide comprehensive insights into the causal agent of *Ilex verticillata* leaf blight and the host’s physiological and molecular responses to *Alternaria alternata* infection. Our results confirm *A. alternata* as the primary pathogen responsible for leaf blight in *I. verticillata*, aligning with previous reports of *Alternaria* species causing similar diseases in other plants, such as watermelon and maize [7,8,9]. The pathogen’s identification was supported by robust morphological and molecular evidence, including phylogenetic analysis of ITS, TEF1-α, G3PDH, and RPB2 loci, which placed our isolates within a well-supported clade of *A. alternata* reference strains. This consistency underscores the reliability of our identification and highlights the pathogen’s adaptability across diverse hosts. The pathogenicity assays revealed distinct disease progression stages, from early necrotic lesions to advanced tissue degradation, mirroring symptoms observed in natural infections. Notably, the re-isolation of *A. alternata* from symptomatic tissues fulfilled Koch’s postulates, solidifying its role as the causal agent. Interestingly, while *Colletotrichum siamense* has been implicated in *I. verticillata* leaf blight elsewhere [14], its absence in our isolates suggests regional or varietal differences in pathogen prevalence. This discrepancy may arise from environmental factors or host genotype-specific interactions [34,35,36], emphasizing the need for localized pathogen surveys to inform disease management strategies.

The transcriptomic analysis unveiled dynamic reprogramming of *I. verticillata*’s defense mechanisms during infection. Early-stage responses were marked by the upregulation of pattern recognition receptors (PRRs) such as CERK1 and XA21, which are critical for pathogen detection [20,25]. However, as infection progressed, a downregulation of cell wall reinforcement genes was observed. This may reflect either pathogen-induced manipulation of host defenses or a host-driven resource reallocation strategy (a classic growth-defense trade-off). The alignment of this pattern with findings in rice blast interactions [37,38,39] is significant, as it suggests that suppression of host cell wall defenses may be a conserved strategy employed by diverse fungal pathogens. Furthermore, the sustained upregulation of antioxidant enzymes like SOD and CAT indicates a sustained activation of the host’s antioxidant system to mitigate oxidative stress, a common theme in plant-pathogen interactions [40]. The antioxidant defense system in *I. verticillata* exhibited a nuanced response to *A. alternata* infection. The significant increase in SOD activity alongside the suppression of POD activity suggests a distinct management of reactive oxygen species (ROS). This specific pattern, contrasting with reports in other pathosystems [41], could be particularly advantageous for the necrotrophic lifestyle of *A. alternata*; the sustained oxidative burst may promote host cell death, which facilitates nutrient acquisition by the pathogen. The early surge in MDA content indicated severe oxidative membrane damage, consistent with reports in gardenia [25,40]. However, the subsequent decline in MDA levels, coupled with sustained CAT activity, suggests activation of repair mechanisms or adaptive responses to prolonged stress. These findings highlight the delicate balance between oxidative damage and defense in *I. verticillata*, with implications for breeding more resilient cultivars.

Phytohormone profiling revealed dynamic and coordinated changes between stress and growth-related hormones during infection. The biphasic response of ABA, initial decline followed by partial recovery, mirrors its dual role in stress adaptation and growth regulation. Similarly, the transient peak in SA levels at 12 dpi points to its involvement in systemic acquired resistance, while the rebound of JA in late infection stages underscores its centrality in defense [29,31]. The antagonistic dynamics between SA and JA align with their established roles in mediating trade-offs between biotic and abiotic stress responses [28,42]. Notably, the modulation of growth hormones like auxin and gibberellins suggests pathogen exploitation of host growth pathways to facilitate colonization, a strategy observed in other pathosystems [43]. The transcriptional changes in hormone-related genes provide supporting evidence for the observed physiological shifts. The differential expression of key regulators in the JA (e.g., *JAZ*, *MYC2*) and SA (e.g., *PAL*, PR-1) pathways is consistent with the activation of canonical defense signaling. Furthermore, the modulation of ABA biosynthesis and catabolism genes aligns with its suggested role in stress responses [44,45,46]. While the hormone quantification by ELISA indicates relative changes, these trends are corroborated by the congruent transcriptomic data. Collectively, these results suggest a model of a coordinated hormonal network contributing to the defense strategy of *I. verticillata*.

Our findings offer several specific avenues for managing *I. verticillata* leaf blight. The conclusive identification of *A. alternata* as the causal agent immediately informs the selection of effective fungicides known to target this pathogen. Furthermore, the key defense components (such as the early upregulation of the pattern recognition receptor *CERK1*, the sustained induction of SOD and CAT, and the distinct SA/JA hormonal crosstalk) provide concrete targets for molecular breeding. These genes could serve as biomarkers for screening resistant germplasm or be prioritized for genetic engineering. The temporal dynamics of the SA and JA pathways suggest that timed exogenous application of these hormones could potentially potentiate the plant’s innate defense response, a hypothesis that warrants direct experimental testing [44]. Future work should focus on the functional validation of candidate genes like *CERK1* and *WRKY2* through transgenic approaches, and on investigating how the root microbiome influences the observed susceptibility to *A. alternata*. By building directly upon the molecular framework established here, future strategies can be developed to enhance the resilience of this valuable horticultural species.

## 4. Materials and Methods

### 4.1. Plant Material Collection and Preparation

Whorled holly (*Ilex verticillata* ‘Meister’) leaves were collected at 0-, 3-, 12-, and 19 days post-inoculation (dpi) with leaf blight pathogens, along with control leaves treated with sterile potato dextrose agar (PDA) medium. These time points were selected to correspond with distinct stages of disease progression observed in preliminary pathogenicity assays: the early symptom appearance (3 dpi), the active mid-stage (12 dpi), and the advanced tissue degradation phase (19 dpi). All collected samples were immediately flash-frozen in liquid nitrogen and stored at −80 °C until further analysis. The study utilized two-year-old cuttings of *I. verticillata* ‘Meister’, with healthy control plants provided by Hangzhou Runtu Horticulture Technology Co., Ltd (Hangzhou, China). Diseased plant materials were collected from field sites during November 2021 and October 2022 to enhance the representativeness of the pathogen isolates by sampling across different seasons (autumn) and years.

### 4.2. Experimental Design and Growth Conditions

The experiment was conducted using two-year-old *Ilex* ‘Meister’ cuttings maintained under controlled greenhouse conditions at Zhejiang Agricultural and Forestry University. Environmental parameters were maintained at 25–28 °C with 70–80% relative humidity throughout the study period. Plants were divided into two treatment groups: (1) control plants inoculated with sterile PDA medium and (2) experimental plants inoculated with purified pathogen strains. The inoculation procedure involved placing mycelial plugs (5 mm diameter) from actively growing cultures onto surface-sterilized leaves, with appropriate controls to validate the pathogenicity tests. Following inoculation, disease progression was monitored regularly, and leaf samples were collected at predetermined intervals for subsequent biochemical and molecular analyses.

### 4.3. Pathogen Isolation and Culture

Fungal pathogens were isolated from symptomatic leaf tissues collected at the junction of diseased and healthy areas. Tissue sections (0.5 × 0.5 cm) were surface sterilized in 75% ethanol for 1 min, followed by three rinses with sterile distilled water. The sterilized tissues were aseptically transferred to PDA plates and incubated at 28 °C for 2–3 days. Pure cultures were obtained through hyphal-tip subculturing onto fresh PDA medium. Three morphologically distinct isolates (designated Y9-2-1, Y10, and Y16) were selected for further characterization and maintained as stock cultures on PDA slants at 4 °C for short-term storage and in 20% glycerol at −80 °C for long-term preservation.

### 4.4. Morphological Characterization and Molecular Identification

Isolates were cultured on PDA at 28 °C for 4–6 days. Colony morphology (color, texture, growth pattern) was documented photographically. Spore structures were examined microscopically using the slide culture technique: sterile slides were inserted at 45° angles into PDA plates, allowing hyphal growth along the slide surface for direct microscopic observation. Microscopic examination was carried out using an upright microscope (Olympus BX53, Shanghai Lai’s Electronic Technology Co., Ltd. [Shanghai, China]) at magnifications of 100×, 400×, and 1000× (using a 100× oil immersion objective lens for 1000× magnification). The identification of fungal structures (conidiophores, conidia) was based on standard taxonomic keys for *Alternaria* species, with key diagnostic criteria including: the morphology and coloration of conidiophores; and for conidia, their size, shape, the presence of both transverse and longitudinal septa, and their formation in chains.

For DNA extraction, fungal mycelium (100 mg) was ground in liquid nitrogen and processed using a modified CTAB protocol. Samples were lysed in CTAB buffer (with β-mercaptoethanol) at 65 °C for 20 min, followed by chloroform:isoamyl alcohol (24:1) extraction and isopropanol precipitation. DNA pellets were washed with 75% ethanol, air-dried, and resuspended in nuclease-free water. Quality was verified via spectrophotometry and 1.2% agarose gel electrophoresis. For phylogenetic identification of Alternaria isolates, a standard multi-locus sequence analysis (MLSA) approach was employed, targeting four genetic loci: the internal transcribed spacer (*ITS*) region, and portions of the translation elongation factor 1-alpha (*TEF1-α*), glyceraldehyde-3-phosphate dehydrogenase (*G3PDH*), and RNA polymerase II second largest subunit (*RPB2*) genes. This combination provides a balance between universal applicability (ITS) and higher phylogenetic resolution from protein-coding genes. These four loci were amplified (PCR conditions: 95 °C/5 min; 35 cycles of 95 °C/30 s, 55 °C/30 s, 72 °C/30 s; 72 °C/5 min). Primer sequences are listed in Table 1.

For phylogenetic analysis sequences were aligned against NCBI references using MEGA7.0. Concatenated datasets were analyzed in PhyloSuite v1.2.2, with Bayesian inference (MrBayes) and neighbor-joining (NJ) methods (1000 bootstrap replicates). Clades with ≥50% support were considered significant.

### 4.5. Pathogenicity Assays

For in vitro detached leaf assay, mycelial plugs (5 mm) from 7-day-old cultures were placed on surface-sterilized leaves wounded with sterile needles. Leaves were incubated on moist filter paper in Petri dishes (28 °C, darkness). Controls received sterile PDA plugs. Disease progression was monitored for 7 days, with re-isolation performed to fulfill Koch’s postulates.

In vivo inoculation of potted plants was inoculated as above, with inoculated sites wrapped in sterile water-moistened cotton and parafilm. Plants were maintained at 25–28 °C, 80% RH under natural light cycles. Symptom development was recorded photographically.

### 4.6. Transcriptome Sequencing and Bioinformatics Analysis

Total RNA was extracted from infected and control leaves (three biological replicates per time point) by Biomike Biotechnology Co., Ltd. (Tianjin, China) and performed Illumina-based sequencing. cDNA libraries were prepared following quality checks, where RNA integrity was confirmed using an Agilent 2100 Bioanalyzer (Agilent Technologies Co., Ltd. [Beijing, China]; RNA Integrity Number, RIN > 8.0) and sequenced on the Illumina platform. Raw reads were processed to remove adapters and low-quality sequences, yielding clean data. Clean reads were aligned to the *I. verticillata* using StringTie and gene expression levels were quantified as FPKM (Fragments Per Kilobase of transcript per Million mapped reads). FPKM expression values of all genes are represented in Supplementary Table S1. Functional annotation was performed via alignment against GO (Gene Ontology) and KEGG (Kyoto Encyclopedia of Genes and Genomes) databases. Differentially expressed genes (DEGs) were identified using DESeq2 with thresholds of false discovery rate (FDR) < 0.05 and |log2Fold Change| ≥ 1. Functional annotation used GO and KEGG databases via Biomike Cloud Platform (www.biocloud.net, accessed on 23 September 2023).

### 4.7. Determination of Endogenous Plant Hormones

The levels of endogenous hormones were quantified using commercial enzyme-linked immunosorbent assay (ELISA) kits by Shanghai Fanke Industrial Co., Ltd. (Shanghai, China) according to the manufacturer’s instructions. The specific catalog numbers for the kits used were as follows: abscisic acid (ABA): F4924-A; jasmonic acid (JA): F4976-A; salicylic acid (SA): F7911-A. Briefly, frozen leaf tissues were homogenized in the specific extraction buffer provided with each respective kit. The supernatant was collected after centrifugation. For the standard curves, the series of standard solutions provided in each kit were used, following the kit’s instructions. Absorbance was measured using a microplate reader, and hormone concentrations in the samples were interpolated from their corresponding standard curves. Raw hormone values were represented in Supplementary Table S2.

### 4.8. Extraction of Crude Enzyme Solution

For antioxidant defense enzyme assays, approximately 0.2 g of powdered leaf tissue was homogenized in 2 mL of ice-cold 50 mM phosphate-buffered saline (PBS, pH 7.8) containing 0.2 mM EDTA and polyvinylpolypyrrolidone (PVPP). The homogenate was centrifuged at 12,000 rpm for 20 min at 4 °C, and the supernatant was collected as a crude enzyme extract.

#### 4.8.1. Superoxide Dismutase (SOD) Activity Assay

SOD activity was determined by monitoring the inhibition of nitroblue tetrazolium (NBT) reduction. The reaction mixture (1.5 mL total volume) contained 870 μL PBS (pH 7.8), 150 μL each of 130 mM methionine, 750 μM NBT, 100 μM riboflavin, and Na_2_-EDTA, along with 30 μL of crude enzyme extract. Control tubes replaced the enzyme extract with PBS. Samples were exposed to 150 μmol/m^2^·s light for 25–30 min, and absorbance was measured at 560 nm. SOD activity (U·g^−1^·min^−1^) was calculated using the formula:

SOD activity (U·g^−1^·min^−1^) = (*Ack* − *AE*) × *V*/*Ack* × 0.5 × *Fw* × *a*

where *Ack* and *AE* are the absorbances of the control and sample, respectively, *V* is the total volume of crude enzyme extract, *Fw* is the fresh weight of the sample, and *a* is the total reaction volume (1.5 mL).

#### 4.8.2. Catalase (CAT) Activity Assay

CAT activity was measured by tracking the decomposition of H_2_O_2_ at 240 nm. The reaction mixture (1 mL) contained 875 μL PBS (pH 7.0), 100 μL 100 mM H_2_O_2_, and 25 μL crude enzyme extract. The decrease in absorbance was recorded over 30 s. CAT activity (U·g^−1^·min^−1^) was calculated as:

CAT activity (U·g^−1^·min^−1^) = Δ*A* × *Vt*/0.1 × *V*1 × *t* × *Fw*

where Δ*A* is the absorbance change, *Vt* is the total enzyme extract volume, *V*1 is the volume used in the assay, *t* is the reaction time, and *Fw* is the sample fresh weight.

#### 4.8.3. Peroxidase (POD) Activity Assay

POD activity was determined by measuring the oxidation of guaiacol at 470 nm according to the method of Kochba et al. [47] with modifications from Zhang et al. [48]. The reaction mixture (1 mL) consisted of 800 μL PBS (pH 7.0), 50 μL 20 mM H_2_O_2_, 50 μL 1% guaiacol, and 100 μL crude enzyme extract. The increase in absorbance was recorded for 30 s. POD activity was calculated using:

POD activity = (Δ*A* × *Vt*)/(0.001 × *Fw* × vs. × *t*)

where Δ*A* is the absorbance change, *Vt* is the total enzyme volume, vs. is the volume used in the assay, *t* is the reaction time, and *Fw* is the sample fresh weight.

### 4.9. Malondialdehyde (MDA) Content Determination

MDA, a lipid peroxidation marker, was quantified using the thiobarbituric acid (TBA) method. A reaction mixture containing 300 μL 10% trichloroacetic acid (TCA), 100 μL 0.6% TBA, and 100 μL crude enzyme extract was incubated at 95 °C for 30 min, cooled, and centrifuged. Absorbance was measured at 532 nm, 600 nm, and 450 nm. MDA content (μmol/g) was calculated as:

MDA content (μmol/g) = [6.452 × (*A*532 *− A*600) − 0.559 × *A*450] × *V*1/(*Fw* × *V*2)

where *V*1 is the total extract volume, *V*2 is the volume used in the assay, and *Fw* is the sample fresh weight.

### 4.10. Statistical Analysis

All experimental data were analyzed using IBM SPSS Statistics (version 20). For physiological and biochemical measurements, the assumptions of normality (Shapiro–Wilk test) and homogeneity of variances (Levene’s test) were verified prior to analysis. All data met these assumptions (*p* > 0.05). A one-way analysis of variance (ANOVA) was then performed to determine significant differences among treatment groups, followed by Duncan’s multiple range test for post hoc comparisons at a significance level of *p* < 0.05. Three biological replicates were included for each experimental condition to ensure statistical reliability.

Differentially expressed genes (DEGs) were visualized as heatmaps using TBtools software (Version 1.123) [49], with hierarchical clustering based on normalized expression values (FPKM). The color gradient in heatmaps represented relative expression levels, with standardized z-scores used to facilitate comparison across genes and samples [49]. All statistical tests were two-tailed, and data were checked for normality and homogeneity of variance prior to analysis. Results are presented as mean values ± standard deviation (SD) unless otherwise specified.

## 5. Conclusions

This study establishes *Alternaria alternata* as the primary causative agent of leaf blight in *Ilex verticillata*, providing critical insights into the host’s defense mechanisms. The integration of morphological, molecular, and pathogenicity analyses confirmed the pathogen’s identity, while transcriptomic and physiological investigations revealed a multi-layered defense strategy. The host’s response involved dynamic reprogramming of antioxidant systems, phytohormone signaling, and metabolic pathways, highlighting the complexity of its interaction with *A. alternata*. Notably, the antagonistic interplay between salicylic acid and jasmonic acid, along with sustained oxidative stress management, underscores the plant’s adaptive resilience. These findings advance our understanding of *I. verticillata’s* susceptibility to fungal pathogens and offer practical implications for disease management. The specific defense-related components identified in this study such as the early activation of pattern-recognition receptors (e.g., *CERK1*), the sustained antioxidant activity (e.g., SOD, CAT), and the dynamic crosstalk between salicylic acid and jasmonic acid signaling, provide potential targets for breeding resistant cultivars. By bridging fundamental and applied perspectives, this work contributes to the sustainable cultivation of *I. verticillata*, ensuring its ornamental and economic value in horticulture.

## Figures and Tables

**Table 1:** Primers used for identification of *Alternaria*.

Gene	Primer Sequence (5′–3′)
*ITS*	ITS1: TCCGTAGGTGAACCTGCGG
	ITS4: TCCTCCGCTTATTGATATGC
*TEF1-α*	EF1-728F: CATCGAGAAGTTCGAGAAGG
	EF1-986R: TACTTGAAGGAACCCTACC
*G3PDH*	F: ATTGACATCGTCGCTGTCAACGA
	R: ACCCCACTCGTTGTCGTACCA
*RPB2*	F: GATGATCGTGATCATTTCGG
	R: CCCATAGCTTGCTTACCCAT

## Data Availability

Data is contained within the article or Supplementary Materials.

## Supplementary Materials

The following supporting information can be downloaded at: https://www.mdpi.com/article/10.3390/plants14193057/s1, Figure S1: Pathogenicity assessment and microbial community analysis following Koch's postulates. (A) Corresponding purification and re-isolated fungal culture, [Symptoms on detached *Ilex verticillate* leaves inoculated with *Alternaria alternata* isolate Y9-2-1 (Figure 4C,D). (B) Heatmap showing the relative abundance of the dominant fungal genera in the leaf microbial community of control (CK) and *A. alternata*-inoculated (Y9-2-1) leaves. The color intensity indicates the relative abundance from low (blue) to high (red).; Figure S2: DEGs expression model of JA metabolic pathway in *I. verticillata* leaves after infection with *A. alternata*; Table S1: All gene expression; Table S2: Raw Hormone data.
