# Supplementary material for: Leaf Blight in Ilex verticillata Caused by Alternaria alternata: Mechanisms of Antioxidant Defense, Phytohormone Crosstalk, and Oxidative Stress Responses
Source: Plants (Basel). 2025 Oct 3;14(19):3057. doi: 10.3390/plants14193057 (PMC12526423; doi:10.3390/plants14193057)
Supplement: Supplementary file 1 [file plants-14-03057-s001.zip › Supplementary Figures.docx]

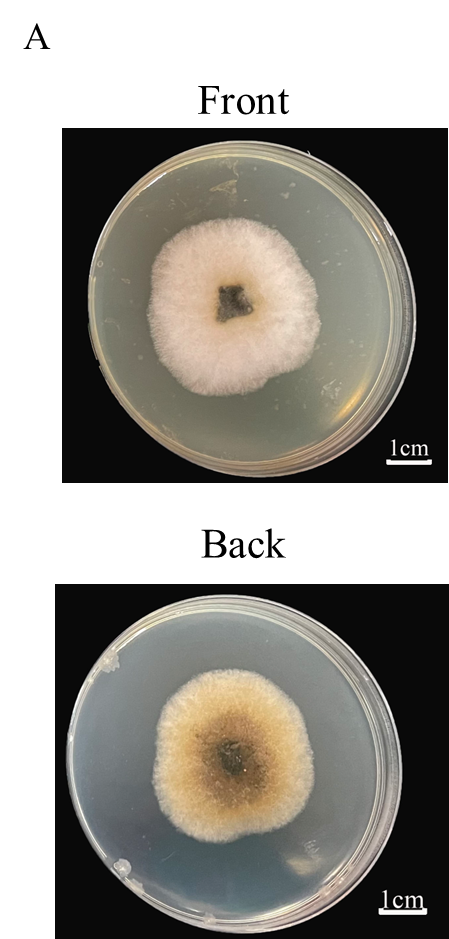

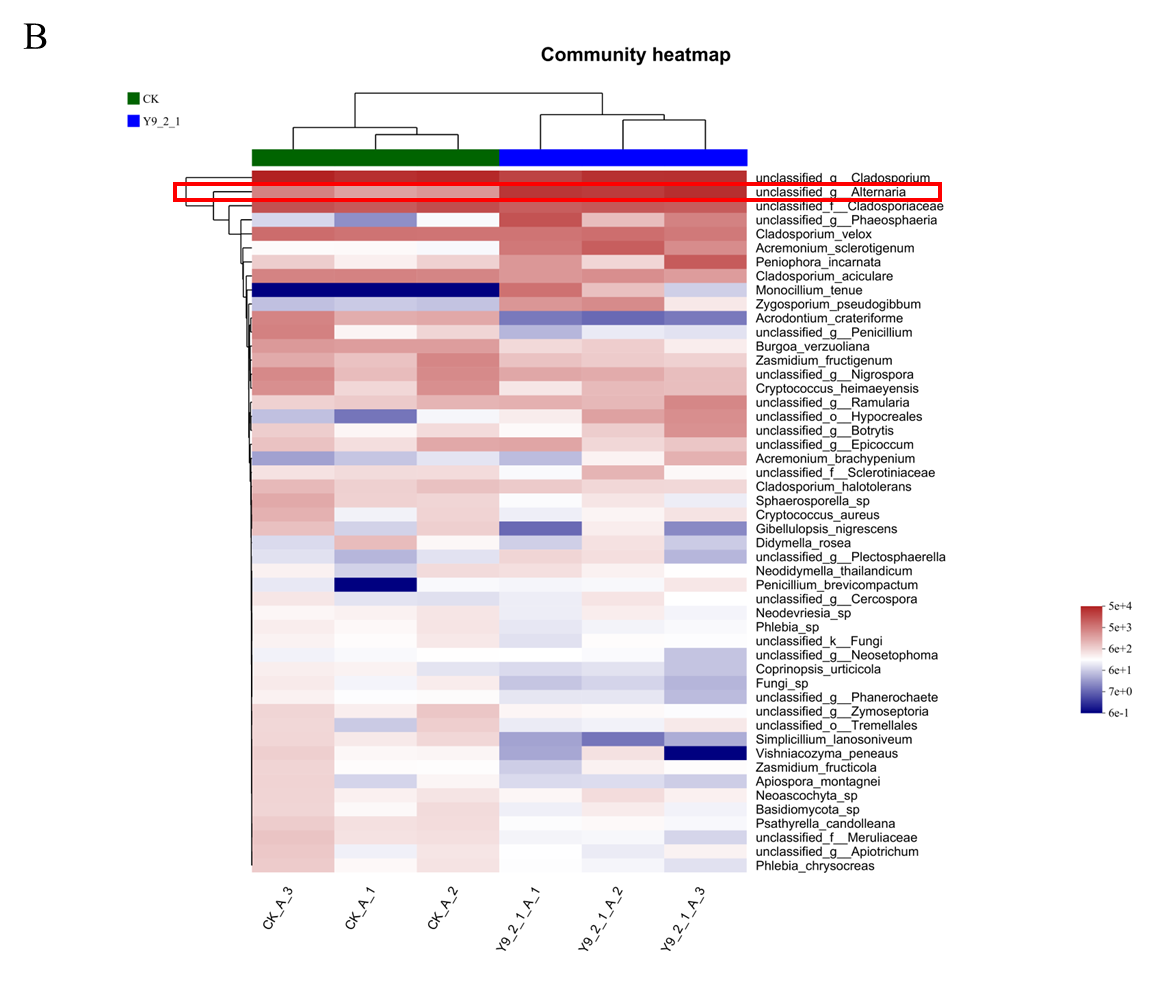


**Supplementary Figure S1:** Pathogenicity assessment and microbial community analysis following Koch's postulates. (A) Corresponding purification and re-isolated fungal culture, [Symptoms on detached *Ilex verticillata* leaves inoculated with *Alternaria alternata* isolate Y9-2-1 (Figure 4C, D). (B) Heatmap showing the relative abundance of the dominant fungal genera in the leaf microbial community of control (CK) and *A. alternata*-inoculated (Y9-2-1) leaves. The color intensity indicates the relative abundance from low (blue) to high (red).

All genes of *LOX2S, AOS*, *AOC* and *OPR and* 3 genes in *OPCL1* were highly expressed in the early stage of infection, and all showed a down-regulated expression trend. In the late stage of infection , *MFP2* gene and *ACAA1* gene were mainly up-regulated (Figure S3).


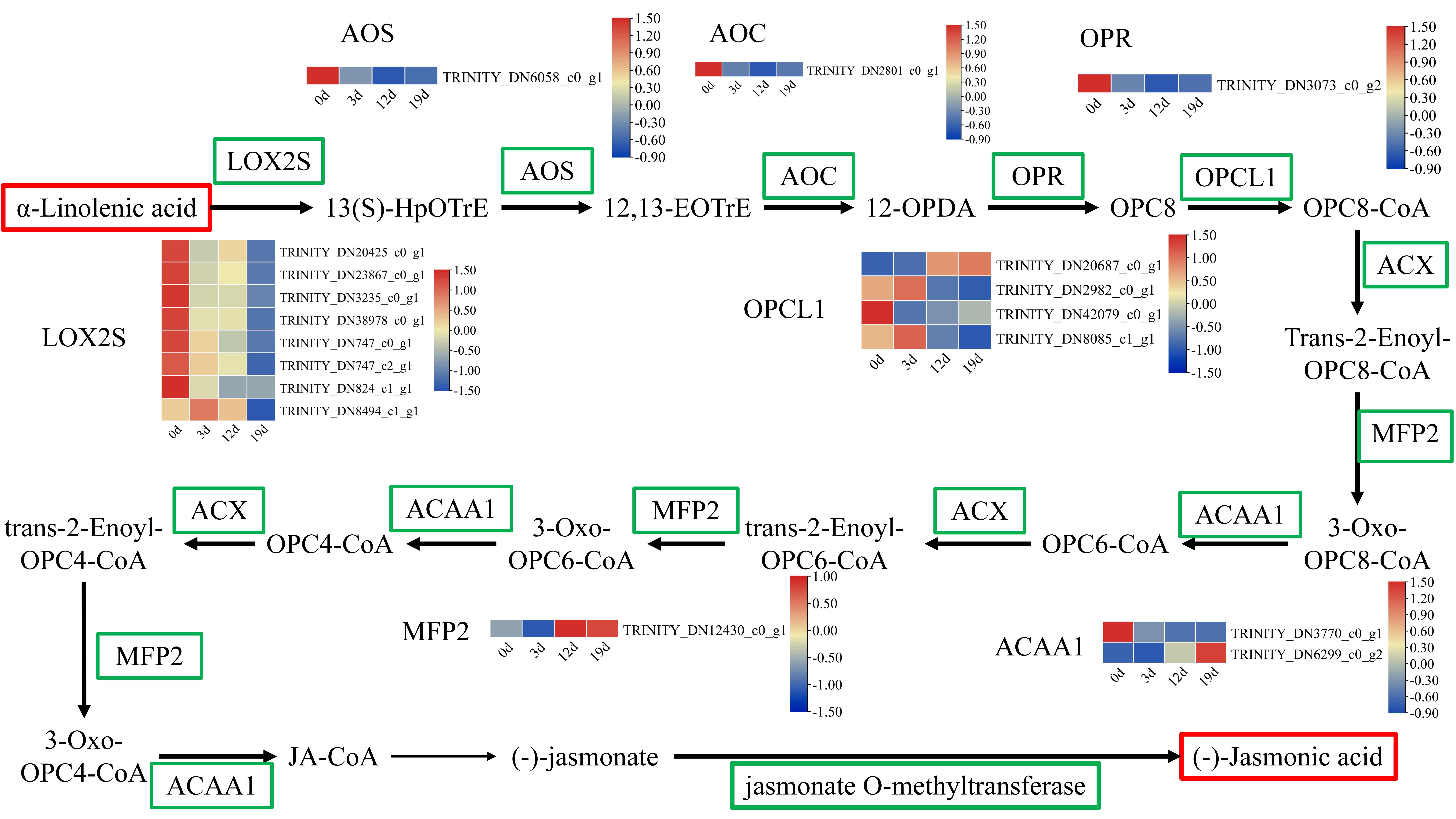


Figure S2: DEGs expression model of JA metabolic pathway in *I. verticillata* leaves after infection with *A. alternata*.
